# Specific synaptopathies diversify brain responses and hearing disorders: you lose the gain from early life

**DOI:** 10.1007/s00441-015-2168-x

**Published:** 2015-04-07

**Authors:** Marlies Knipper, Rama Panford-Walsh, Wibke Singer, Lukas Rüttiger, Ulrike Zimmermann

**Affiliations:** 1 Department of Otolaryngology, Head and Neck Surgery, Tübingen Hearing Research Center (THRC), Molecular Physiology of Hearing, University of Tübingen, Elfriede-Aulhorn-Straße 5, 72076 Tübingen, Germany; 2 DNA Genotek, 2 Beaverbrook Road, Kanata, ON K2K 1L1 Canada

**Keywords:** Inner hair cell, Auditory neuropathy, Tinnitus, Hyperacusis, Auditory cortex

## Abstract

Before hearing onset, inner hair cell (IHC) maturation proceeds under the influence of spontaneous Ca^2+^ action potentials (APs). The temporal signature of the IHC Ca^2+^ AP is modified through an efferent cholinergic feedback from the medial olivocochlear bundle (MOC) and drives the IHC pre- and post-synapse phenotype towards low spontaneous (spike) rate (SR), high-threshold characteristics. With sensory experience, the IHC pre- and post-synapse phenotype matures towards the instruction of low-SR, high-threshold and of high-SR, low-threshold auditory fiber characteristics. Corticosteroid feedback together with local brain-derived nerve growth factor (BDNF) and catecholaminergic neurotransmitters (dopamine) might be essential for this developmental step. In this review, we address the question of whether the control of low-SR and high-SR fiber characteristics is linked to various degrees of vulnerability of auditory fibers in the mature system. In particular, we examine several IHC synaptopathies in the context of various hearing disorders and exemplified shortfalls before and after hearing onset.

## Introduction

During maturation of the first synapse of the auditory system at the inner hair cell (IHC) synapse onto auditory nerve (AN) fibers, huge modifications in electrical and morphological properties occur. Accompanied by spontaneous IHC Ca^2+^ action potentials (APs), AN fibers mature stepwise and develop characteristics with different spontaneous (spike) rates (SR) and different sensitivities (thresholds; Sachs and Abbas 1974; Yates 1991).

Maturation continues with sensory experience under the control of central feedback loops. Within the scope of this review, we summarize the way that feedback circuits from the central nervous system to the IHC synapse might stepwise improve the temporal and frequency resolution of the mature auditory system. We examine the driving forces behind the various auditory fibers of different sensitivities and discuss them in the context of the different degrees of vulnerability of the IHC pre- and post-synapse possibly leading to diverse hearing disorders.

## Critical maturation steps of IHC synapse before hearing onset

### IHC synapse maturation steps during spontaneous Ca^2+^ APs

**Fig. 1:**
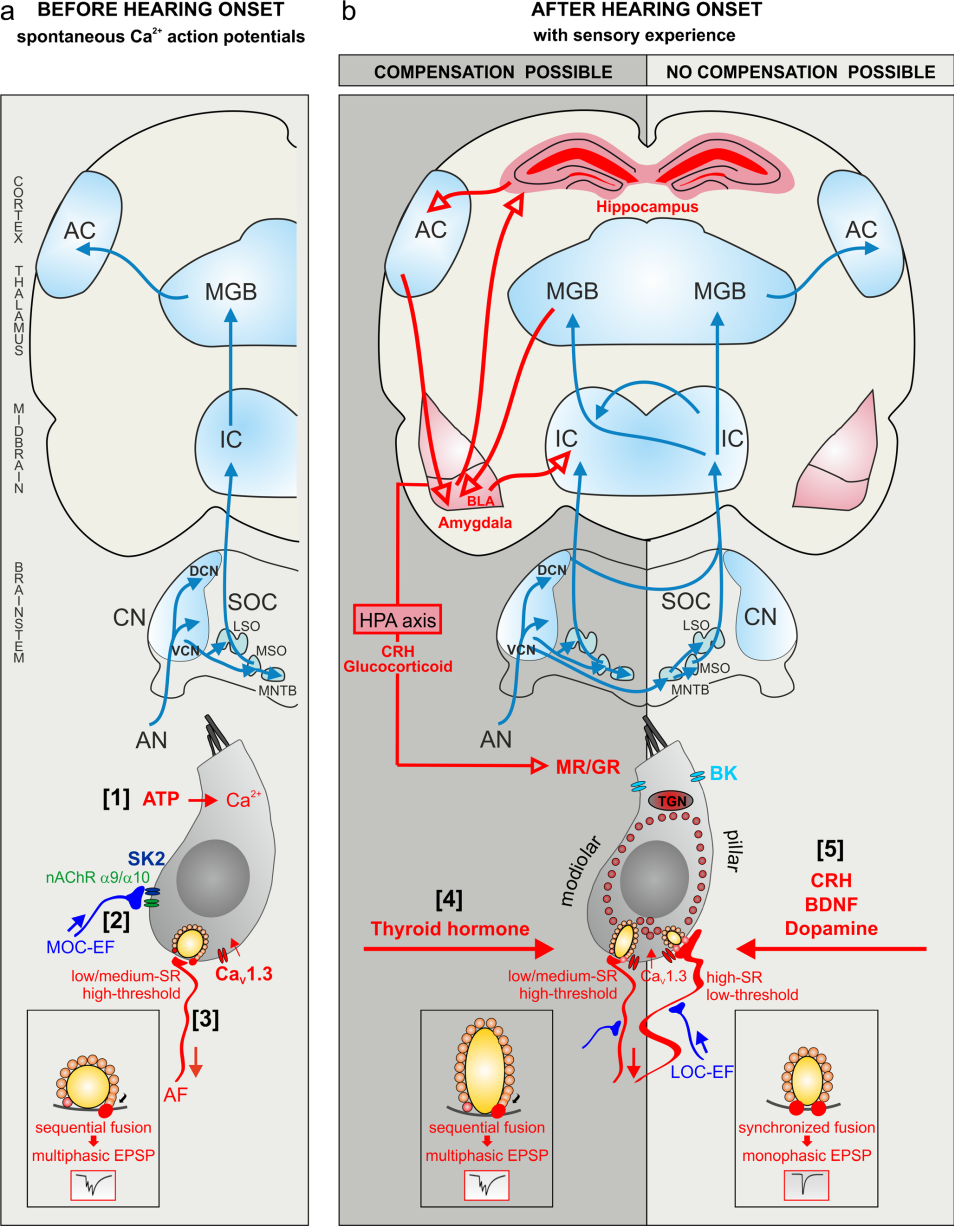
Maturation of the inner hair cell synapse (IHC). **a** Prior to hearing onset, IHCs exhibit spontaneous Ca^2+^ action potentials (APs), which are elicited through ATP signaling (*[1]*). Influenced by the temporal signature provided through nicotinic acetylcholine receptor (*nAChR*)α9/α10-mediated axosomatic IHC input (*[2]*), the synaptic machinery of IHCs progressively evolves and can form low-SR (spontaneous [spike] rates), high-threshold fiber characteristics (*[3]*; *EPSP* excitatory post-synaptic potential, *AF* auditory fiber, *AN* auditory nerve, *AC* auditory cortex, *CN* cochlear nucleus, *DCN* dorsal cochlear nucleus, *LSO* lateral superior olivary nucleus, *MSO* medial superior olivary nucleus, *MGB* medial geniculate body, *MNTB* medial nucleus of the trapezoid body, *SOC* superior olivary complex, *VCN* ventral cochlear nucleus, *IC* inferior colliculus, *MOC-EF* medial olivocochlear efferent fibers, *SK2* potassium channel). **b** Ca^2+^ APs are terminated by thyroid hormone action (*[4]*). After hearing onset, with sensory experience, the IHC synapse matures and high-SR fibers characteristics evolve possibly under the influence of a corticotropin-releasing hormone (*CRH*)/brain-derived nerve growth factor (*BDNF*)/dopamine signaling cascade (*[5]*). Low-SR fibers are preferentially lost with moderate sound and, with age, a deprivation paradigm that possibly can be centrally compensated (*left*). In contrast, a critical loss of high-SR fibers might lead to the obstruction of central compensation (*right*; *BK* large-conductance, voltage- and Ca^2+^-activated potassium channel, *BLA* basolateral complex of the amygdala, *HPA* hypothalamic-pituitary-adrenal axis, *LOC-EF* lateral olivocochlear efferent fiber, *MR/GR* glucocorticoid receptors/mineralocorticoid receptors, *TGN* trans-Golgi network)

Prior to hearing onset, spontaneous Ca^2+^ APs of IHCs are the result of ATP that is released from the organ of Kölliker and that drives small voltage input in IHCs after birth (Tritsch and Bergles 2010; Tritsch et al. 2007; for a review, see Wang and Bergles 2014). ATP possibly activates IHCs through ligand-gated ionotropic P2X receptors or G-protein-coupled metabotropic P2Y receptors, both of which have been reported to induce increases of the intracellular Ca^2+^ concentration ([Ca^2+^]_i_; Harada 2010). During the period of spontaneous Ca^2+^ APs, the synaptic machinery of IHCs progressively evolves from multiple spherical bodies, typical of immature hair cells (Fig. 1a), to more confined active zones anchoring a single ribbon (Sobkowicz et al. 1982; Fig. 1b), namely the sub-micrometer electron-dense structures that tether synaptic vesicles (Zenisek et al. 2004). Various excellent reviews have focused on this early developmental period of the inner ear (Castellano-Munoz and Ricci 2014; Fuchs 2005; Marcotti 2012; Moser et al. 2013; Safieddine et al. 2012; Schmitz 2009). The disturbance of ATP signaling during this time has significant adverse effects on the auditory system. This has been shown by reducing ATP-dependent Ca^2+^ signaling activity in cochlear non-sensory cells, an effect that has been achieved by knocking down the expression of phosphatidylinositol phosphate kinase type 1γ (PIPKIγ), a key enzyme in the generation of phosphatidylinositol 4,5-bisphosphate (Rodriguez et al. 2012). PIPKIγ^+/−^ mice have dramatically elevated hearing thresholds, particularly in response to high-frequency sound. Further studies are essential for an understanding of the profound consequences of this early event in more detail.

### IHC pre-synapse maturation steps under influence of thyroid hormone and cholinergic control of spontaneous Ca^2+^ APs

In the immature state, spontaneous Ca^2+^ APs of IHCs are modified by olivocochlear efferent fibers projecting from the ventromedial aspects of superior olivary complex anlage to the cochlea (MOC fibers), where they “wait” under the IHC region before targeting the outer hair cells (OHCs; for a review, see Simmons 2002). They remain in axosomatic contact with IHCs until they are eliminated and IHCs are innervated mainly by afferent fibers, having few, if any, remaining efferent contacts (Liberman et al. 1990). These transient efferent contacts with IHC somata act through nicotinic cholinergic receptors containing α9 and α10 subunits (Katz et al. 2004) and drive a Ca^2+^ influx to activate Ca^2+^-dependent (SK2-containing) K^+^ channels (Katz et al. 2004; Fig. 1a). Upon deletion of α9 (Johnson et al. 2013b) or SK2 (Johnson et al. 2007), the developmental maturation of IHC exocytosis, which typically leads to a more efficient Ca^2+^-dependent IHC exocytotic process, is prevented (Johnson et al. 2007). Analysis of IHC physiology in SK2 knockout animals has led to the current assumption that the proper temporal precision of the Ca^2+^ APs, as a result of acetylcholine release from the efferent terminals through SK2, drives the maturation of the Ca^2+^-dependent exocytosis process (Johnson et al. 2011, 2013a). Thyroid hormone (TH), largely known to influence hearing through driving timely morphological maturation of the organ of Corti (Christ et al. 2004; Deol 1973; Dettling et al. 2013; Knipper et al. 2000; Vanderschueren-Lodeweyckx et al. 1983; Winter et al. 2009), appears also to initiate a crucial step for the final IHC synapse maturation (Brandt et al. 2007; Sendin et al. 2007; for reviews, see Bulankina and Moser 2012; Marcotti 2012). Accordingly, the down-regulation of Ca^2+^ currents and up-regulation of large conductance Ca^2+^-activated potassium channels (BK) on time (Fig. 1b) under the control of TH terminates the Ca^2+^ AP firing (Brandt et al. 2007; Sendin et al. 2007). Notably, Ca^2+^ AP firing (Brandt et al. 2007) and axosomatic cholinergic inputs (Sendin et al. 2007) persist in the absence of TH, meaning that neither the cholinergic input, nor the spontaneous APs per se, nor their combined activity are the trigger for the improved Ca^2+^ efficiency of the IHC exocytosis.

#### Conclusion

During the period of transient cholinergic input to IHCs, pre-synapses mature. However, the improved Ca^2+^ efficiency of IHC exocytosis might rather be a maturation step of the IHC synapse, which is initiated through the elimination of transient axosomatic efferent fibers and the termination of Ca^2+^ AP firing under the control of TH.

### IHC post-synapse maturation steps under thyroid hormone and cholinergic control of spontaneous Ca^2+^ APs

During the period of spontaneous Ca^2+^-dependent APs of IHCs, modified by efferent fibers and TH, low-sensitive auditory fiber characteristics appear to dominate. In the mature cochlea, AN fibers receiving signals from a single IHC via a single ribbon synapse (Liberman 1980b; Spoendlin 1969) can be grouped into two main categories according to their SR: low- and medium-SR fibers (<0.5 to 18 spikes/s) and high-SR fibers (>18 spikes/s; Sachs and Abbas 1974; Yates 1991). High-SR fibers are sensitive to low-sound pressure levels, whereas low-SR fibers have a threshold elevated by about 20–40 dB (Sachs and Abbas 1974; Yates 1991).

When recording from distal afferent AN fibers in the vicinity of IHC synapses before and after hearing onset, the rise time and amplitudes of the excitatory post-synaptic currents (EPSCs) develop only over time (Grant et al. 2010, 2011). Prior to hearing onset, EPSCs are multiphasic, resulting from uncoordinated multivesicular release (Glowatzki and Fuchs 2002). These multiphasic EPSCs are postulated to occur in response to transmitter release from larger elongated ribbons located at the modiolar side of IHCs (Liberman et al. 2011; Yin et al. 2014; Fig. 1b, left). The IHC vesicle fusion steps that generate low EPSC amplitude distribution have been speculated to generate low-SR, high-threshold AN characteristics (Grant et al. 2010). On the basis of this assumption, low-SR, high-threshold characteristics would occur at the time when IHCs still generate spontaneous Ca^2+^ APs under the influence of transient axosomatic cholinergic input (Fig. 1a).

#### Conclusion and open questions

At the time of cholinergic axosomatic input to IHC synapses, possibly low- or medium-SR, high-threshold AN characteristics appear to predominate. As discussed above, TH drives the termination of Ca^2+^ APs, including the elimination of cholinergic axosomatic efferent contacts. Whether TH initiates the maturation of Ca^2+^ efficacy of exocytosis through the termination of the Ca^2+^ AP-dependent step (Fig. 1a, b) remains unknown.

### IHC synapse maturation steps that drive through spontaneous Ca^2+^ APs central processes

The Ca^2+^ currents that drive the spontaneous activity in the cochlea before hearing onset are carried by L-type Ca_V_1.3 channels. They define output activity that is derived from the cochlea and that is transmitted along the central auditory pathway (for reviews, see Tritsch and Bergles 2010; Tritsch et al. 2007). Several studies have analyzed the influence of the spontaneous activity patterns of the immature cochlea on the maturation of central processes. Spontaneous Ca^2+^ APs generated by immature IHCs have been suggested as a prerequisite for neuron survival and the generation of topographically ordered connections in the auditory pathway. More details of this topic are given, for example, by Clause et al. (2014), Friauf and Lohmann (1999), Hirtz et al. (2011), Kandler et al. (2009) and Kandler and Gillespie (2005). Less clear is the way that the temporal signature of the spontaneous AP pattern, dictated by cholinergic input on IHCs (Johnson et al. 2013a; Sendin et al. 2014), influences the maturation of the central auditory pathway. In this context, we can learn from the visual system in which spontaneous retinal Ca^2+^ waves are also under cholinergic control prior to eye opening (Stellwagen and Shatz 2002; Wong et al. 1995; for a review, see Blankenship and Feller 2010). Here, during the maturation of the retina, cholinergic starburst amacrine cells initiate waves of propagating excitation that drive neighboring retinal ganglion cells (RGCs) to fire spatiotemporally correlated bursts of APs (Speer et al. 2010; Wong et al. 1995). The findings indicate that the precise timing of the post-synaptic spike pattern of RGCs and its synchrony is the result of the cholinergic (amacrine) influence on burst activity and the most critical step for successful eye-specific retinogeniculate segregation (Speer et al. 2014). Moreover, this precise timing of the activity of three outputs is a prerequisite for spike-dependent central plasticity, as shown in frogs for the retinotectal pathway driven by visual experience (Mu and Poo 2006).

#### Open questions

The cholinergic IHC input defines the temporal signature of Ca^2+^ APs of IHCs (Clause et al. 2014; Johnson et al. 2013a; Sendin et al. 2014). An open question is whether this “gained” temporal precision of spiral ganglion neuron (SGN) output through cholinergic feedback on IHCs defines the segregation of binaural hearing cues and thereby would establish the precondition of spike-dependent central plasticity changes driven by auditory experience (Fig. 1). Future studies should test the role of cholinergic feedback on IHCs, e.g., through spiral ganglion-specific deletion of acetylcholine receptor α9/α10 subunits by using the *Cre*-lox system.

## Critical maturation steps of IHC synapse after hearing onset

### IHC pre-synapse maturation steps with auditory experience

Hearing onset (later than postnatal day 12 [>P12] in rodents) and the first sound-driven brainstem responses (Corwin et al. 1982) are initiated through three processes. (1) TH-induced elimination of efferent cholinergic contacts (Sendin et al. 2007), (2) TH-driven up-regulation of BK currents and (3) TH-dependent decline of Ca^2+^ currents, all of which contribute to the termination of Ca^2+^ APs in IHCs (Brandt et al. 2007). This is also the period when tighter spatial coupling between Ca^2+^ influx and exocytosis in IHCs is reported (Marcotti 2012) and when Ca_V_1.3 channels form stripe-like clusters rather than the smaller round clusters present in immature IHCs (Wong et al. 2014). At this time, the expression of otoferlin, a C2-domain protein, shown to be essential for IHC exocytosis by acting as a Ca^2+^ sensor in vesicle fusion (Ramakrishnan et al. 2009; Roux et al. 2006), is also up-regulated (Beurg et al. 2010; Roux et al. 2006). Elevation of otoferlin expression in IHCs occurs under the control of TH (Johnson et al. 2010), a step that thus probably depends on the elimination of axosomatic efferent fibers and the termination of Ca^2+^ APs. Evidence is increasing that otoferlin is critical for the replenishment of synaptic vesicles after hearing onset (Johnson et al. 2010), a process that may go hand in hand with the binding of otoferlin to myosin VI (Heidrych et al. 2008) and the adaptor protein complex 2 (AP-2; Duncker et al. 2013). Both proteins are assumed to drive a clathrin-mediated endocytosis process (for reviews, see Hirst and Robinson 1998; Tumbarello et al. 2013). Reduced AP-2 (Duncker et al. 2013) and otoferlin (Johnson et al. 2010) levels are observed in the absence of TH, together with a failure to determine the high-order Ca^2+^ dependence of neurotransmitter release and a decline of vesicle replenishment through the secondary releasable pool (Johnson et al. 2010). This means that TH, through the up-regulation of AP-2 and otoferlin or promotion of AP-2−otoferlin interaction, can influence an efficient coupling of exocytosis to endocytosis (Duncker et al. 2013). Indeed, such a coupling in mature IHCs has been suggested to be attributable to a Ca^2+^-regulated vesicle resupply by endocytic events that might sustain the indefatigable release properties of mature IHCs (Griesinger et al. 2005; Levic et al. 2011; Schnee et al. 2011). A challenge for the future will be to test whether the coupling of the Ca^2+^-sensing otoferlin to the adaptor protein complex AP-2 is preferentially achieved at active zones of high-SR, low-threshold fibers to assure a maximal resupply of vesicles of these early saturating auditory fibers, even at lowest sound-pressure levels.

#### Conclusion

The possibility of the efficient Ca^2+^-dependent coupling of exocytosis and endocytosis in IHC synapses after hearing onset needs to be reconsidered and regarded in the context of an improved sound sensitivity carried out by high-SR, low-threshold fibers.

### IHC post-synapse maturation steps with auditory experience

Immediately following the onset of hearing, excitatory post-synaptic potentials with sharp rise times and monoexponential decays (monophasic) develop and probably represent the highly synchronous fusion of many vesicles (Grant et al. 2010). They are probably activated through glutamate release from small compact spherical ribbons in IHC synapses located at the pillar side of IHCs (Fig. 1b, right; Liberman 1982; Yin et al. 2014). Accordingly, from prior to hearing (shown for rats at P8−P11) to just past the onset of hearing (P19−P21), the average median EPSC amplitude more than doubles and the mode of the amplitude distribution increases more than ten times (Grant et al. 2010). This significantly higher proportion of larger monophasic EPSCs found in IHC post-synapses of hearing rodents (Grant et al. 2010) probably contributes through their high discharge rate to the compound action potential (CAP) threshold of the AN (Bourien et al. 2014). As low-SR fibers do not contribute to the threshold of CAPs (Bourien et al. 2014), we thus have to conclude that, after hearing onset, CAP thresholds are expected to improve coincident with the establishment of high-SR fibers. We can speculate that the high-SR fiber-driven sound responses after hearing onset are the driving force for the tighter coupling of exocytosis and endocytosis in the auditory brainstem after hearing onset (Midorikawa et al. 2014), in comparison with before hearing onset.

#### Conclusion and open questions

On the pre-synaptic side of the IHC synapse, after hearing onset, Ca^2+^ dependency of exocytosis becomes more efficient and the coupling of exocytosis and endocytosis is enhanced. This occurs when a progression of a higher proportion of auditory fibers with high-SR, low-threshold characteristics is established on the post-synaptic IHC side. Two questions remain to be answered: (1) what is the trigger of this crucial process and (2) how are these final pre- and post-synaptic IHC maturation events interconnected? The following sections provide some currently discussed ideas cocnerning the central feedback control of the IHC maturation steps with sensory experience.

### IHC synapse maturation steps with sensory experience control cortical maturation

The IHC pre- and post-synaptic maturation that occurs after hearing onset and that leads to the establishment of lower threshold sound responses has to be seen as a critical, behaviorally relevant step for any individual species. Accordingly, high-SR AN fibers define the lowest thresholds and narrowest dynamic ranges at their characteristic frequency (CF) as shown in many species, including rats, mice and gerbils (el Barbary 1991; Ohlemiller and Echteler 1990; Taberner and Liberman 2005). As, at any given CF in the auditory system, high-SR neurons have the shortest latencies and low-SR neurons have the longest latencies (Rhode and Smith 1986), high-SR, low-threshold fibers possibly define behaviorally relevant response thresholds along the entire auditory pathway. This has, however, not yet been established. Until recently, sound detection during quiescence has been suggested to be mediated by a long-term integration process in the central auditory system (see, e.g., Eddins and Green 1995). Newer findings suggest that inter-aural (Zohar et al. 2011) and perceptional thresholds might be defined by onset stimuli levels (Heil et al. 2008; Meddis 2006) and thus might be influenced by the specialized IHC synaptic ribbons. IHC ribbons define the precision of spike rates and thereby possibly the temporal precision of sound stimuli at its onset (Buran et al. 2010). Here, the high-SR fibers that define CF thresholds and respond to sound with the lowest latencies have been suggested as candidate fibers for defining perceptional thresholds (Heil et al. 2008; Meddis 2006). The significant increase of fibers that probably exhibit high-SR, low-threshold characteristics after the onset of hearing (Grant et al. 2010) might therefore be linked to central processes known to depend on sensory experience. In this context, we should note that the final maturation step at the IHC synapse (as the first synapse in the auditory system) goes hand in hand with the accomplishment of the spatial and temporal resolution in the central auditory system (Bureš et al. 2014). Thus, the improved (cortical) resolution is dependent on the first auditory experience in a process that triggers long-lasting inhibitory potentiation within the part of the auditory cortex that receives direct input from the thalamus (Xu et al. 2010). This process critically depends on BDNF, released in the cortex by pyramidal neurons as demonstrated by the inhibition of this process using BDNF scavengers (Xu et al. 2010). In the visual system (Huang et al. 1999; Hubel and Wiesel 1962; Wiesel and Hubel 1963) and somatosensory system (Jiao et al. 2011), sensory experience drives a BDNF-dependent period of increased intra-cortical inhibition (for a review, see Singer et al. 2014). For sensory organs, the functional deficits resulting from the impairment of this crucial developmental step lead (1) to compromised cortical visual receptive field sharpening and to compromised sound processing in the auditory system (Froemke et al. 2007; Kotak et al. 2012; Xu et al. 2010), (2) to loss of visual acuity with subsequent development of amblyopia (lazy eye) in the visual system (Wiesel and Hubel 1963), or (3) to impaired refinement of cortical somatosensory maps in the somatosensory system (Stryker 1978; Wiesel and Hubel 1974).

#### Conclusion and open questions

The final IHC maturation step that coincides with a higher proportion of high-SR, low-threshold auditory fibers immediately after hearing onset has to be regarded in the context of the coincident occurrence of the elevation of intra-cortical inhibition and improved cortical receptive field sharpening. Whether and how both these processes are associated remains unknown. Possible top-down mediators of the crucial IHC synapse maturation steps are given in the next section.

### Possible influence of early environmental enrichment on IHC synapse maturation steps

In the auditory and visual systems, evidence exists that environmental enrichment (EE) can mimic, accelerate, or improve cortical and sub-cortical maturation steps occurring with sensory experience. This is shown in the auditory system in the improved directional sensitivity of primary auditory cortical neurons by EE (Zhang et al. 2009) and in the improved frequency discrimination in the auditory cortex and improved sensitivity to quiet sounds by EE (Bose et al. 2010; Cai et al. 2009; Centanni et al. 2013; Engineer et al. 2004). Moreover, in the sub-cortical auditory neurons of the inferior colliculus (IC), previous studies have demonstrated an improved excitation threshold and an improved intensity and frequency resolution through early postnatal EE (Bureš et al. 2014). In the visual system, successful dendritic segregation and improved visual acuity have been shown by EE even up to P10, before the eyes are opened (Landi et al. 2007a, 2007b).

#### Conclusion and open questions

The findings of an improvement in central auditory processing subsequent to early EE suggest an early central top-down signal to the cochlea, one that modulates IHC pre- and post-synaptic events independently of sensory experience. Candidate triggers for this process are discussed in the following.

### Influence of cochlear BDNF and dopamine on final IHC synapse maturation steps

In the visual system, a reduction of BDNF expression in RGCs of EE-exposed animals prior to eye opening has been shown to counteract EE effects on retinal and cortical acuity (Landi et al. 2007a, 2007b), emphasizing that EE influences cortical processes through a BDNF-dependent step in the retina. The target receptors of BDNF in RGCs prior to eye opening are suggested to be TrkB receptors found in retinal dopaminergic amacrine interneurons (Landi et al. 2007a, 2007b, 2009). The way in which BDNF in RGCs can alter visual acuity through dopamine-induced effects is still unknown. Here, BDNF- and dopamine-induced improvement of retinal acuity through receptive field re-organization of RGCs (Sinclair et al. 2004; Witkovsky 2004) have been considered (Landi et al. 2009).

In the auditory system, cochlear BDNF and dopamine have been shown to influence hearing sensitivity. Accordingly, the Pax2-*Cre*-induced excision of the BDNF gene (BDNF^*Pax2*^ knockout) leading to BDNF deletion in preferentially the cochlea, dorsal cochlear nucleus and IC induces significantly reduced exocytosis activity in the high-frequency cochlear turns (Zuccotti et al. 2012). The reduced exocytosis goes hand in hand with the reduced number of IHC ribbons in otherwise normally matured IHCs (Zuccotti et al. 2012). Auditory brainstem response (ABR) amplitudes of summed AN activity, as measured through supra-threshold auditory brainstem responses, generated at the level of the AN (ABR wave I) and IC (ABR wave IV), are significantly reduced in BDNF^*Pax2*^ knockout mice. This happens independently of the electromechanical amplification from OHCs as measured by the distortion products of otoacoustic emission (Zuccotti et al. 2012). BDNF in the cochlea has been concluded to drive late IHC pre- and post-synaptic maturation processes probably of auditory fibers that define hearing thresholds (Zuccotti et al. 2012). This hypothesis has been confirmed through extracellular recording of sound responses in the IC of BDNF^*Pax2*^ knockout mice, which demonstrate elevated response thresholds and prolonged latency in IC neurons at high frequencies (unpublished findings). The failure to improve hearing thresholds in the absence of BDNF goes hand in hand with the prevention of the loss of sound sensitivity after acoustic trauma (Zuccotti et al. 2012). This suggests that you cannot lose sensitivity in the mature system if you never have gained it during early development.

Not only cochlear BDNF deletion but also the inhibition of cochlear dopamine activities alter sound sensitivity, mainly independently of OHC function (Ruel et al. 2001). Reduced tonic dopaminergic inhibition of afferent AN fibers by the lateral olivocochlear (LOC) system has been found to elevate CAP thresholds (Ruel et al. 2001). As low-SR fibers do not contribute to CAP thresholds, the observed CAP threshold elevation after the disruption of the dopaminergic lateral efferent pathway (Bourien et al. 2014) must be attributable to the loss of the dopaminergic inhibition of high-SR fibers (Fig. 1b, LOC-EF).

#### Conclusion and open questions

Inhibition or deletion of cochlear BDNF or dopamine function prevents the improvement of sound thresholds after hearing onset, independently of the OHC function. The way in which dopamine alters the response thresholds of afferent fibers after hearing onset remains to be clarified. Here, a dopamine-induced modification of gamma aminobutyric acid A receptor-mediated tonic inhibition can be considered (Crunelli and Di Giovanni 2014).

### Influence of CRH and glucocorticoids on cochlear BDNF- and dopamine-driven IHC synapse maturation steps

The up-regulation of BDNF in the cochlea potentially influencing SGN output activity through possible dopaminergic efferent feedback as described above (Fig. 1b, LOC-EF) should be seen in the context of a top-down influence from the hypothalamus. Accordingly, BDNF and glucocorticoids (cortisol in most mammals including humans; corticosterone in rats and mice) have been shown to regulate directly corticotropin-releasing hormone (CRH) homeostasis in the hypothalamus (Jeanneteau and Chao 2013). BDNF is expressed in the hypothalamus from embryonic day 17 (E17) onwards (Baquet et al. 2004), in the cochlea at P4 and in ascending auditory pathways at >P6-P8 (for a review, see Singer et al. 2014). Thus, early central BDNF influences on CRH (Jeanneteau and Chao 2013) greatly precede the time of the described up-regulation of BDNF in the cochlea at around P4 (Schimmang et al. 2003; Singer et al. 2014; Sobkowicz et al. 2002; Wiechers et al. 1999). Previous findings have shown receptors for CRH (CRF1R) in the cochlea itself (Graham and Vetter 2011). Impaired hearing threshold in CRF1R mutant mice, independent of OHC function, indicate that high-SR, low-threshold responses are controlled by CRH (Graham and Vetter 2011). Given that the CRF1R has been shown to up-regulate BDNF mRNA levels robustly, for example, in the cerebellum (Bayatti et al. 2005), CRH/glucocorticoid effects on cochlear BDNF can be considered as a putative top-down signal that drives improved sound processing with sensory experience (Fig. 1b, CRH, BDNF).

#### Conclusion and open questions

An early influence of CRH and glucocorticoid in the cochlea (Graham and Vetter 2011) on IHC maturation can be considered to promote BDNF (Zuccotti et al. 2012) and dopamine (Ruel et al. 2001) effects, both of which are important for improving sound sensitivity with sensory experience (Fig. 1b). The detailed relationship of these processes needs to be investigated in more detail in future studies. We next challenge the hypothesis that these early differential maturation steps at the IHC pre- and post-synapses before and after hearing onset are linked to various levels of auditory fiber vulnerability in response to noise damage in the adult system.

## Diverse auditory neuropathies linked to different hearing disorders

### IHC synaptopathy and loss of low-SR fibers

Various observations emphasize that substantial AN loss can co-exist with normal hearing thresholds and even with unchanged CAP amplitudes (Bourien et al. 2014) as long as only low-SR fibers are affected (Bourien et al. 2014; Furman et al. 2013; Heinz et al. 2005; Heinz and Young 2004; Kujawa and Liberman 2009; Ruel et al. 2008). This type of neuropathy has been termed “hidden hearing loss” (Schaette and McAlpine 2011), because it does not affect the threshold audiogram. The neuropathy nevertheless becomes obvious in sound-induced supra-threshold amplitudes of cochlear neural responses as a reduction of supra-threshold ABR wave I (Kujawa and Liberman 2009). The consequences of the loss of these fibers for hearing can only be understood when regarding the function of these fibers in more detail. Accordingly, low-SR, high-threshold fibers are important for hearing in noise, since sensitivity is still high, whereas high-SR fibers will be driven into saturation (Bharadwaj et al. 2014; Costalupes et al. 1984). Moreover, the temporal fine structure cues of sound that are important for speech intelligibility in noise (Lorenzi and Moore 2008) and envelope cues that are important for speech-on-speech masking release (Christiansen et al. 2013) rely particularly on low-SR supra-threshold coding. Only these low-SR fibers are still sensitive to fluctuations in sound at comfortable listening levels (for a review, see Bharadwaj et al. 2014). These low-SR auditory fibers are apparently also preferentially lost with age (Sergeyenko et al. 2013) and may explain the finding that aging people experience increasing difficulties of hearing in noise well before any loss of hair cells (Bharadwaj et al. 2014; Dubno et al. 1984; Makary et al. 2011). The loss of low-SR fibers is currently linked to glutamate excitotoxicity after noise and the lower degree of clearance of released glutamate close to these fibers (for reviews, see Moser et al. 2013; Wang and Puel 2008). This reduced degree of clearance of released glutamate is linked to a difference in proteins involved in the clearance of glutamate, such as the glutamate-aspartate transporter (GLAST), which has been found to be less strong on the modiolar side of IHCs, where most low-SR fibers make contacts (Furness and Lawton 2003). Consequently, a significant neural threshold shift and delaying recovery from hearing loss have been observed after acoustic overstimulation upon antagonization of GLAST (Chen et al. 2010; Glowatzki et al. 2006). Moreover, the lower number of mitochondria in low-SR fibers is suggested to contribute to their high sensitivity to metabolic fatigue (for a review, see Bharadwaj et al. 2014). Evidence for this concept has only recently come to light and shows that the distinct auditory fiber pools differ in their sensibility to ouabain, which impairs the Na^+^/K^+^ ATPase transporter, rendering low-SR fibers highly sensitive for ouabain (Bourien et al. 2014). After moderate noise trauma or with age, not only low-SR fibers are lost but also a permanent loss of IHC ribbons occurs (Bharadwaj et al. 2014; Furman et al. 2013; Jaumann et al. 2012; Kujawa and Liberman 2009; Lin et al. 2011; Rüttiger et al. 2013; Zuccotti et al. 2012). Strikingly, whether the loss of AN fibers after acoustic trauma occurs primary (Kujawa and Liberman 2009; Lin et al. 2011) or secondary (Sheets et al. 2012) to IHC ribbon loss is still not known. What appears certain, however, is that neither noise-induced nor kainate-induced swelling is seen in the terminals of type II fibers contacting OHCs (Pujol and Puel 1999). Type II terminals also do not express the same AMPA type glutamate receptors (e.g., GluA2) as do the type I terminals (Liberman et al. 2011). Therefore, glutamate excitotoxicity is currently assumed to be a pathology restricted to the IHC area and not to the OHC area (for reviews, see Bharadwaj et al. 2014; Moser et al. 2013; Wang and Puel 2008). What also appears to be certain is that, gradually over time and secondary to the degeneration of the afferent dendrites of AN fibers, spiral ganglion cells undergo neurodegeneration, as shown after glutamate-induced excitotoxic trauma in vitro (Puel et al. 2002; Wang and Green 2011) and in vivo after intense tone exposure (Godfrey et al. 2012) or after long-term mild trauma (Lin et al. 2011).

#### Conclusion and open questions

Preferential vulnerability to neurodegeneration is present in the dendrites of low-SR, high-threshold fibers in response to noise and aging and can co-exist with a normal hearing threshold and unchanged CAP amplitudes. Whether this dendritic neurodegeneration occurs primary or secondary to the damage of the IHC synapse remains to be clarified. Whether delayed spiral ganglion cell loss secondary to AN degeneration is preferentially restricted to neurons with low-SR, high-threshold characteristics is also unknown.

### IHC synaptopathy and loss of high-SR fibers

High-SR, low-threshold fibers determine the ability to detect sounds in a quiet environment but are so sensitive to sound that the discharge rate saturates as early as about 20–30 dB above threshold (Yates 1991). Continuous activation by noise causes rapid synaptic fatigue of high-SR fibers through vesicle depletion (Bharadwaj et al. 2014; Costalupes 1985; Costalupes et al. 1984). The higher levels of mitochondria (Liberman 1982) and GLAST proteins (Furness and Lawton 2003) at the pillar side, where high-SR fibers predominate, might prevent vulnerability to excitotoxic events (Liberman 1982). Interestly, this protection from metabolic fatigue can be considered as an evolutionary adaptation for maintaining high sensitivity to sound and thereby the behaviorally relevant response thresholds along the entire auditory pathway. On the other hand, pressure to reduce the vulnerability of high-SR fibers might also come from a possible role that this fiber type plays in homeostatic adaptation processes following peripheral injury or even plasticity events in general. Accordingly, the persistence of a critical level of high-SR, low-threshold fibers after noise trauma has been predicted as a prerequisite for the generation of a sufficient increase in discharge rate in auditory fibers targeting neurons in the brainstem, as calculated in a computational model (Schaette and Kempter 2009, 2012). Searching for a correlate for tinnitus, which is assumed to correlate with central hyperactivity, the model predicts a loss of low-SR, high-threshold fibers as a neural correlate of tinnitus (Schaette and McAlpine 2011). Indeed, only a few studies have found a critical loss of high-SR, low-threshold fibers after noise trauma. Interestingly, in contrast to Schaette and McAlpine (2011), other authors have suggested not low-SR but rather high-SR fiber loss as a correlate of tinnitus. This was first described through the demonstration of a deafferentation of large-diameter auditory fibers with morphological characteristics of high-SR fibers or through a critical IHC ribbon loss in a tinnitus behavioral animal model (Bauer et al. 2007; Rüttiger et al. 2013; Singer et al. 2013).

#### Conclusion and open questions

After noise exposure, a deafferentation of IHCs (IHC synaptopathy) possibly comprises low-SR, high-threshold and high-SR, low-threshold fibers. We discuss, in the following section, the possibility of a differential loss of these fiber types in hearing disorders such as tinnitus and hyperacusis.

### Differential auditory fiber loss in tinnitus and hyperacusis

A differential AN fiber loss has previously been hypothesized to be linked in various ways to the central responsiveness related to diverse auditory disorders such as tinnitus and hyperacusis (Knipper et al. 2013). Tinnitus is a disorder of perception of phantom sound that is also known as ringing in the ear or head. Tinnitus affects 10−20 % of the general population. An estimated 50 million people in the United States suffer from chronic tinnitus persisting for longer than 6 months. Hyperacusis is a disorder of loudness perception, in which sound intensities that are considered comfortable by most people are perceived to be unbearably loud (Baguley 2003). With an approximate prevalence of about 10−15 % of the population (Gilles et al. 2012), the prevalence of hyperacusis is similar to that of tinnitus (Shargorodsky et al. 2010). Although hearing loss is a major risk factor for tinnitus and hyperacusis, profound evidence has meanwhile been presented showing that both tinnitus and hyperacusis can occur with (clinically) normal auditory thresholds (Geven et al. 2011; Gu et al. 2010; Kim et al. 2011; Langers et al. 2012; Lockwood et al. 2002; Roberts et al. 2010; Saunders 2007; Shiomi et al. 1997; Tan et al. 2013; Weisz et al. 2006; Zeng 2013). As shown in animal studies, this is linked to the deafferentation of IHCs (Bauer et al. 2007; Rüttiger et al. 2013; Singer et al. 2013; Tan et al. 2013; Weisz et al. 2006; for reviews, see Euteneuer and Praetorius 2014; Hébert et al. 2013; Knipper et al. 2010, 2012, 2013).

**Fig. 2:**
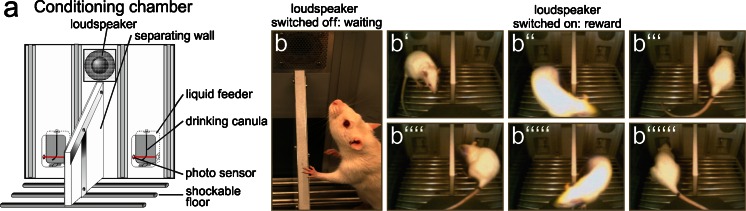
Animal behavior model that enables rats that perceive tinnitus to be distinguished from those that do not. **a** Representation of a rat behavior box (conditioning chamber). **b** Rats are trained to sit on a platform during silence (**b**) and to move in order to receive sugar water rewards during sound (**b’−b’’’’’’**). When animals perceive tinnitus, they move to obtain a sugar reward despite silence. For details, see Rüttiger et al. (2003)

**Fig. 3:**
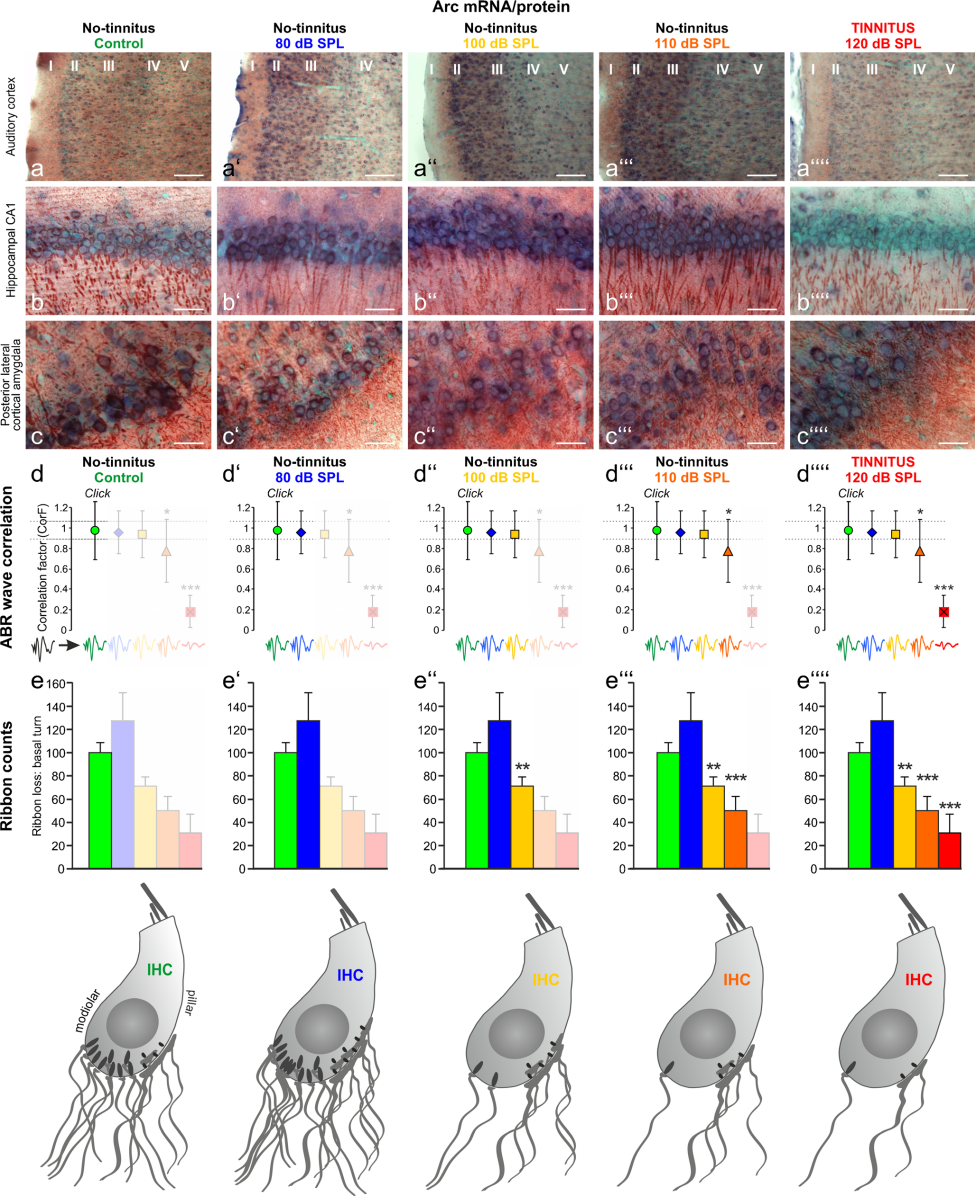
Association of central responsiveness and hearing disorder to various degrees of IHC neurodegeneration. Activity-dependent plasticity gene *Arc*/*Arg3.1* expression (*blue* Arc/Arg3.1 mRNA, *red* Arc/Arg.31 protein) in individual adult female Wistar rats not exposed (control) or exposed to 80, 100, 110 and 120 dB SPL (sound pressure level) at 14 days following sound exposure. **a–a’’’’** Auditory cortex. **b–b’’’’** Hippocampal CA1 region. Note that Arc/Arg3.1 expression in tinnitus-free animals (*No-tinnitus*, *80*, *100*, *110 dB SPL*) is gradually enhanced, whereas in tinnitus animals (*TINNITUS*, *120 dB SPL*), no change in the expression of Arc/Arg3.1 is observed. **c–c’’’’** Arc/Arg3.1 expression levels in the posterior-lateral region of the cortical amygdala, used as a control tissue, remain unaltered. **d–d’’’’** Auditory brainstem response (ABR) wave functions of individual animals not exposed (control) or exposed to 80, 100, 110 and 120 dB SPL at 14 days following sound exposure (*black wave* in **d** *below graph* average ABR wave function of untreated animals). The changes in waveforms and signals were calculated as a correlation factor (CorF) as described in Singer et al. (2013). *Dashed lines* indicate the 95 % confidence interval for the controls. Note that auditory cortex and hippocampal Arc/Arg3.1 expression is correlated with ABR waves. **e–e’’’’** Counts of IHC ribbons revealed a gradual reduction in the basal cochlear turn of animals exposed to 100, 110 and 120 dB SPL. For more information, see Singer et al. (2013). Note that auditory cortex and hippocampal Arc/Arg3.1 expression is also correlated with IHC ribbon counts of the basal cochlear turn. Interestingly, mobilized Arc/Arg3.1 levels and restored ABR waves occur despite increasing ribbon loss after exposure to 100 and 110 dB SPL sound in *No-tinnitus* animals. In contrast, a critical loss of IHC ribbons after 120 dB SPL noise exposure was linked with a failure to mobilize Arc/Arg3.1 and restore ABR waves in *TINNITUS* animals. For further details, see Singer et al. (2013)

Interestingly, depending on the degree of IHC deafferentation, a surprisingly divergent recovery rate of supra-threshold delayed ABR waves and central responsiveness, as measured through activity-dependent genes such as Arc/Arg3.1, can be observed in behaviorally trained animals (Fig. 2; Knipper et al. 2013; Rüttiger et al. 2013; Singer et al. 2013). Behavioral studies on rats can be performed with validated conditioning in which animals are trained to sit on a platform during silence (Fig. 2b) but to move to obtain sugar water rewards during sound (Fig. 2b’−b’’’’’’; Rüttiger et al. 2003). Arc/Arg3.1 is a cytoskeletal protein that is mobilized after long-term potentiation (LTP)-like activity to scale AMPA receptors in post-synapses up and down, a process essential for LTP consolidation. This process is also a prerequisite for long-term increases in the strength of a synapse in response to a reduced firing rate (Beique et al. 2011) or to visual deprivation (Gao et al. 2010; for reviews, see Bramham et al. 2008, 2010; Korb and Finkbeiner 2011; Tzingounis and Nicoll 2006). Accordingly, the findings in rats show that the expression levels of Arc/Arg 3.1 are gradually increased in layer II−III of the auditory cortex (Fig. 3a−a’’’) and in the hippocampal CA1 region (Fig. 3b−b’’’) at 2–3 weeks after acoustically exposing animals to 80, 100, or 110 dB SPL (sound pressure level) sound for 2 h (Singer et al. 2013), despite decreasing numbers of IHC ribbons (Fig. 3e−e’’’). Moreover, as long as Arc/Arg3.1 expression is increasingly mobilized, a successful restoration of centrally generated ABR waves (ABR wave IV) occurs (Fig. 3d−d’’’) always being correlated with No-Tinnitus (Fig. 3, No-tinnitus). When IHC ribbons are critically declined, as observed after 120 dB SPL sound exposure, Arc/Arg 3.1 mobilization drops out and central ABR waves fail to be restored and are now linked to tinnitus (Fig. 3, Tinnitus, 120 dB SPL; Singer et al. 2013). A contrasting central brain response dependent on the degree of IHC ribbon loss occurs, even among animals that are equally acoustically exposed to moderate acoustic trauma (Rüttiger et al. 2013). At 2 to 3 weeks after a moderate acoustic trauma, either a lower IHC ribbon loss is correlated with the successful restoration of ABR wave IV in animals without tinnitus or a higher degree of deafferentation and IHC ribbon loss is correlated with an inability to restore ABR wave IV with behaviorally tested tinnitus (Rüttiger et al. 2013). As seen before, animals without tinnitus exhibit higher cortical Arc/Arg3.1 expression levels than those with tinnitus (Singer et al. 2013). The vulnerability of IHC synapses to moderate acoustic trauma is enhanced when corticosteroid levels in animals are strongly elevated through a stress paradigm at the time of trauma (Singer et al. 2013).

#### Conclusion and open questions

To date, few studies indicate a differential central responsiveness following a different degree of deafferentation of IHCs. A moderate degree of deafferentation (low-SR, high-threshold fibers) permits central mobilization of the activity-related gene Arc/Arg3.1 linked with restored supra-threshold ABR waves in No-tinnitus animals. A higher degree of deafferentation (high-SR, low-threshold fibers) obstructs compensation of central network activity in tinnitus animals (see Fig. 1b). If, indeed, a differential low-SR and high-SR auditory fiber loss distinguishes No-tinnitus from tinnitus animals, single auditory fiber recording might be required. Moreover, the role of afferent type II fibers projecting to OHCs as a candidate driving force for altering central responsiveness has to be further investigated. More important is to clarify the question as to which individual conditions favor a selective IHC synapse vulnerability for either the low-SR or high-SR fiber type, despite similar traumatic exposure paradigms.

## Possible modulators of differential noise vulnerability at IHC level

### Modulators of vulnerability of low-SR, high-threshold IHC responses

As contributors to preferential damage of low-SR, high-threshold fibers, *N*-methyl-D-aspartate (NMDA) receptors, which are predominantly located on the modiolar side of IHCs, should also be considered (Pujol et al. 1992). NMDA receptors appear to predominate at sides where a higher percentage of LOC efferent fibers seems to terminate on low-SR fibers (Liberman 1980a). Both kainate and NMDA receptor subunits (NR1 and NR2A-D) have been described on the afferent post-synaptic density (for a review, see Puel et al. 2002). In the adult system, an increase in glutamate release from IHCs has been shown to activate NMDA receptors and to trigger excessive Ca^2+^ in SGN dendrites (Sanchez et al. 2014). The resulting over-excitation increases ATP demand and correspondingly increases reactive oxygen species at IHC-SGN synapses (Sahley et al. 2013). Excessive Ca^2+^ influx through NMDA receptors therefore results in a cascade of metabolic events including free-radical production, protease and endonuclease activation and eventually neuronal death (Parsons and Raymond 2014). Thus, NMDA receptor activation could contribute to the excitotoxic events that preferentially lead to trauma- or age-dependent loss of low-SR fibers (see “IHC synaptopathy and loss of low-SR fibers”). In agreement with this, in the presence of NMDA receptor antagonists, acute insult induces a smaller decrease in hearing sensitivity, faster recovery from loss and a smaller reduction in surface AMPA receptor expression (Chen et al. 2007).

**Fig. 4:**
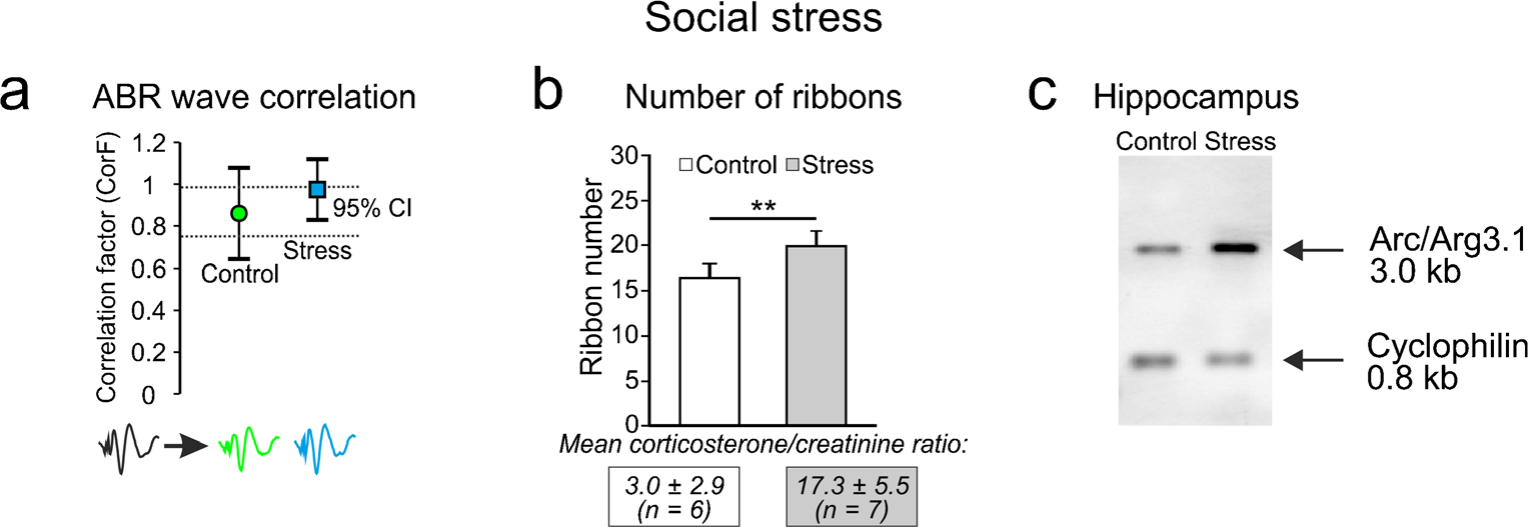
Stress priming affects ABR waveforms and IHC ribbon numbers. **a** Averaged ABR wave functions before and 2 days after stress priming. Waveforms of stressed animals did not significantly differ from ABR waveforms of control animals, as indicated by a similar CorF but variability was smaller. **b** Number of IHC ribbons was significantly increased by 22 % in stressed animals. **c** Northern blot analysis for Arc/Arg3.1 mRNA shows that stress priming alone increased Arc/Arg3.1 mRNA levels in the hippocampus. The housekeeping gene cyclophilin was used as a loading control (for further details, see Singer et al. 2013)

We should also consider a possible endogenous protective potential of selected corticosteroid activities of low-SR, high-threshold fibers for the following reasons. Glucocorticoid receptors or mineralocorticoid receptors are expressed in hair cells and the spiral ganglion (Terakado et al. 2011; Yao and Rarey 1996). IHC pre- and post-synapses might thus be the target of elevated cortisol levels whenever the hypothalamic-pituitary-adrenal system triggered by CRH is activated and corticosteroid hormone is secreted by the adrenal glands (De Kloet et al. 1998). Elevated corticosteroid levels triggered by a social stressor paradigm have been shown to induce a long-lasting reduction in ABR wave variations pointing to a strongly consolidated synaptic connection (Fig. 4a), a slightly increased IHC ribbon number (Fig. 4b) and increased expression of the plasticity gene Arc/Arg3.1 in the hippocampus (Fig. 4c; Singer et al. 2013). This indicates that corticosteroid activities can stabilize the auditory circuit activity that also involves, via amygdaloid output projections, hippocampal activity (for a review, see Canlon et al. 2013). Moreover, as stated above, a moderate corticosteroid level is correlated with a higher maintenance of ABR waves after noise exposure (indicating better recovery of ABR waveform), a less pronounced loss of IHC ribbons in high-frequency cochlear turns and more markedly elevated Arc/Arg3.1 levels in the hippocampus (Singer et al. 2013). Endogenous glucocorticoid activities can thus be considered to stabilize the IHC low-SR fiber contacts under traumatic conditions.

#### Open questions

LOC efferent fibers contacting low-SR, high-threshold fibers should be revisited in future studies in the context of NMDA-receptor- or glucocorticoid-induced changes in the vulnerability of these fibers. Future therapeutic intervention of diseases related to a loss of low-SR, high-threshold fibers, as is presbyacusis or hyperacusis, should consider these mediators.

### Modulators of high-SR, low-threshold IHC responses

A more severe loss of ribbons in high-frequency cochlear turns in tinnitus animals (Rüttiger et al. 2013; Singer et al. 2013) has been linked to an abnormally large ribbon size and a reduction of the basolateral length of IHCs in these cochlear turns (Knipper et al. 2013; Rüttiger et al. 2013). With regard to this event in the context of the development of the IHC synapse during hearing onset, a reduction in the basolateral length of IHCs with abnormally large ribbons interestingly resembles the IHC phenotype of mutant mice with myosin VI or otoferlin deletion (Heidrych et al. 2009; Johnson et al. 2010). The abnormal ribbon size in these mutants has been hypothesized to be linked to a disturbed membrane cycling process (Heidrych et al. 2008). The establishment of a possible Ca^2+^-dependent coupling of exocytosis and endocytosis through otoferlin−AP-2 interaction is predicted to occur after hearing onset (Duncker et al. 2013), coincident with the time when high-SR fiber characteristics are established (see “IHC post-synapse maturation steps with auditory experience”). We urgently need to consider modulators of this late IHC synapse maturation period in the context of a defined vulnerability of high-SR, low-threshold fibers and the subsequent altered central responsiveness. If we predict, as stated above (“Possible influence of early environmental enrichment on IHC synapse maturation steps”), a role of BDNF- and dopamine signaling subsequent to CRH and glucocorticoid receptors as a driving force for improved sound signaling, we have to consider these factors as being candidate mediators involved in changing the vulnerability of a loss of these IHC pre- and post-synaptic elements in the adult system (Knipper et al. 2013; Zuccotti et al. 2012).

#### Open questions

Further studies are necessary to identify the mechanism of high-SR, low-threshold AN vulnerability in more detail. This is of particular interest in the context of the urgent need for the development of improved causal therapies for diseases linked with this defined AN fiber loss, as is possibly tinnitus.

## Concluding remarks and future perspectives

In a hypothetical and simplified model, the following maturation steps at the pre- and post-synapse of afferent and efferent fibers at the IHC level may subsequently occur: (1) before hearing onset, the temporal signature of a defined cholinergic feedback control on IHC Ca^2+^ APs drives low-SR, high-threshold auditory fiber characteristics; (2) the temporal signature of these spontaneous IHC bursts might serve as the driving force for the segregation of binaural cues; (3) TH eliminates cholinergic input to IHCs and terminates spontaneous APs carried by Ca^2+^ APs, a prerequisite for the subsequent maturation of efficient Ca^2+^-dependent exocytosis and/or efficient Ca^2+^-dependent coupling of exocytosis and endocytosis; (4) CRH-, BDNF- and dopamine-dependent steps influence the properties of high-SR auditory fibers with low-thresholds (Ruel et al. 2006); (5) this process appears to improve sound sensitivity (signal-to-noise ratio) and central auditory resolution, with future studies being essential to investigate to what extent this improved sound sensitivity also improves the signal-to-noise ratio of the efferent feedback to OHCs; (6) to this end, the differential vulnerability of low- and high-SR fiber types for injury in the adult system, leading to different central responsiveness and hearing disorders, have to be seen in the context of the de-maturation of feedback control loops.

## Abbreviations

ABR: Auditory brainstem response
AN: Auditory nerve
AP: Action potential
AP-2: Adaptor protein complex 2
BDNF: Brain-derived nerve growth factor
BK: Large-conductance, voltage- and Ca^2+^-activated potassium channel
CAP: Compound action potential
CF: Characteristic frequency
CRF1R: Corticotropin-releasing hormone receptor
CRH: Corticotropin-releasing hormone
EE: Environmental enrichment
EPSC: Excitatory post-synaptic current
GLAST: Glutamate-aspartate transporter
IC: Inferior colliculus
IHC: Inner hair cell
LOC: Lateral olivocochlear (bundle)
LTP: Long-term potentiation
MOC: Medial olivocochlear (bundle)
NMDA: *N*-methyl-D-aspartate
OHC: Outer hair cell
PIPKIγ: Phosphatidylinositol phosphate kinase type 1γ
RGC: Retinal ganglion cell
SGN: Spiral ganglion neuron
SPL: Sound pressure level
SR: Spontaneous (discharge) rate
TH: Thyroid hormone
